# Cultivating Multidisciplinarity: Manufacturing and Sensing Challenges in Cultured Meat Production

**DOI:** 10.3390/biology10030204

**Published:** 2021-03-09

**Authors:** Mila Djisalov, Teodora Knežić, Ivana Podunavac, Kristina Živojević, Vasa Radonic, Nikola Ž. Knežević, Ivan Bobrinetskiy, Ivana Gadjanski

**Affiliations:** BioSense Institute, University of Novi Sad, Dr Zorana Djindjica 1, 21000 Novi Sad, Serbia; mila.djisalov@biosense.rs (M.Dj.); teodora.knezic@biosense.rs (T.K.); ivana.podunavac@biosense.rs (I.P.); kristina.zivojevic@biosense.rs (K.Ž.); vasarad@biosense.rs (V.R.); nknezevic@biosense.rs (N.Ž.K.); bobrinet@biosense.rs (I.B.)

**Keywords:** cultured meat, cultivated meat, cellular agriculture, sensors, monitoring, bioreactor, modeling, electrochemical biosensor, photonics

## Abstract

**Simple Summary:**

United Nations estimates that by the year 2050, the population of nearly 10 billion people will have 70% higher food demands than the current food systems can provide for. This needs to be observed in the context of the on-going climate change and related negative effects of the traditional agriculture. Conventional livestock-based value chains contribute to the high greenhouse gas emissions. Meat cultivation via cellular agriculture holds great promise as a method for future food production. Theoretically, it is an ideal way of meat production, humane to the animals and sustainable for the environment, while keeping the same taste and nutritional values as traditional meat. However, in practice, there is still a number of challenges such as large-scale production, regulatory compliance and consumer acceptance. To address these challenges a multidisciplinary approach is necessary. In this optic, we present an overview of the sensor monitoring options for the most relevant parameters for cultured meat bioprocess. Various examples of the sensors to potentially apply in cultured meat production are provided, as well as the options for their integration into different types of bioreactors. Furthermore, we briefly present the current status of the cultured meat research and regulation, societal aspects and its commercialization.

**Abstract:**

Meat cultivation via cellular agriculture holds great promise as a method for future food production. In theory, it is an ideal way of meat production, humane to the animals and sustainable for the environment, while keeping the same taste and nutritional values as traditional meat and having additional benefits such as controlled fat content and absence of antibiotics and hormones used in the traditional meat industry. However, in practice, there is still a number of challenges, such as those associated with the upscale of cultured meat (CM). CM food safety monitoring is a necessary factor when envisioning both the regulatory compliance and consumer acceptance. To achieve this, a multidisciplinary approach is necessary. This includes extensive development of the sensitive and specific analytical devices i.e., sensors to enable reliable food safety monitoring throughout the whole future food supply chain. In addition, advanced monitoring options can help in the further optimization of the meat cultivation which may reduce the currently still high costs of production. This review presents an overview of the sensor monitoring options for the most relevant parameters of importance for meat cultivation. Examples of the various types of sensors that can potentially be used in CM production are provided and the options for their integration into bioreactors, as well as suggestions on further improvements and more advanced integration approaches. In favor of the multidisciplinary approach, we also include an overview of the bioreactor types, scaffolding options as well as imaging techniques relevant for CM research. Furthermore, we briefly present the current status of the CM research and related regulation, societal aspects and challenges to its upscaling and commercialization.

## 1. Cellular Agriculture

According to estimates by the Food and Agriculture Organization of the United Nations (FAO), by the year 2050, the population of nearly 10 billion people will have 70% higher food demands than the current food systems can provide for [1]. This challenge needs to be observed in the context of the on-going climate change and the effects of the traditional agriculture which are contributing to it, in quite a negative way [2].

Traditional livestock-based value chains contribute to the high greenhouse gas (GHG) emissions [3,4], particularly from the ruminants sector [5].

The cultured meat-based value chain would hypothetically allow for lower GHG emissions per unit of produced meat, by avoiding the direct emissions of methane (CH_4_) from enteric fermentation in ruminants, as well as the emissions of both CH_4_ and nitrous oxide (N_2_O) from the animals’ feces and manure. In addition, a cultured meat (CM) supply chain could reduce the land- and water-use footprint of the meat industry [6]. Some of the initial life cycle assessment (LCA) studies seem to confirm such assumptions, showing lower GHG emissions, land requirements and water use for CM production, in comparison to conventional livestock farming [7]. More recent LCA studies provide more detailed insights and highlight the still existing challenges in CM production, such as high-energy demands occurring in the upscaling attempts of the CM cultivation [8,9].

At this point, it is safe to say that further optimization of the CM bioprocess is necessary in order to fulfill potential positive environmental benefits over conventionally produced meat. One way to achieve better efficiency of the CM bioprocess is via improving sensing abilities, for the reasons discussed in this review.

However, climate-related effects are not the only impact CM may exert. Agriculture, particularly massive-scale animal farming, causes a habitat disturbance for many wildlife species. A recent study by Gibb et al. showed that disrupted habitats have a greater proportion of species that host zoonotic diseases than those in undisturbed areas [10]. This is particularly important in view of the on-going pandemic of SARS-CoV-2 virus [11] and other recent outbreaks of other zoonotic viruses such as H5N1 and H7N9 avian flu [12].

Another important aspect is related to the increased antimicrobial resistance (AMR) in livestock, detected in the past 20 years. This is a direct consequence of the wide use of antimicrobials in intensive animal production systems [13], which may be reduced via the switch to CM production. In order to prevent contamination, the standard cell culture procedures routinely use antibiotics and fungicides, albeit in significantly smaller quantities when compared to livestock farming. However, this may not even be necessary for CM, as recent studies show that antibiotics-free cultivation procedures for CM may even be more favorable for the serum-free media adaptation [14,15]. A CM bioprocess that could be implemented without the use of antibiotics and with serum-free media would be considered as a double positive effect, since the major proportion of the costs of CM production comes from the serum- and media-related costs [16].

Taking into account the described negative consequences of conventional livestock-based meat production, it can be concluded that humanity needs to re-focus on more sustainable and safer ways of food production. This is where alternative protein research and cellular agriculture (CA) in particular come to focus [17,18,19].

In the last several years, CA has become quite a prominent notion in the scientific world as one of the alternative ways of food production. CA is a field of agriculture that involves the manufacturing of products from cell cultures. It is divided into two main areas: (a) **Fermentation-based CA**—which uses engineered microbes to produce recombinant proteins and flavored compounds, and the final product does not contain host cells—and (b) **Tissue-engineering based CA**—which has cells and tissues as the final products [20].

CA, particularly meat cultivation, is expected to positively contribute to the health of human population, in terms of the absence of contaminants and antibiotics during cultivated meat production [21] in addition to the lower incidence rate of foodborne illness and reduction of obesity and cardiovascular diseases, thanks to the more controlled quantity and type of fat in CM [22].

However, since these are still nascent fields of research, there is a substantial need for more extensive development concerning the food safety of such “novel foods” such as CA-generated CM (also known as cultivated meat, cell-based meat, lab-grown meat or in vitro meat) [20]. The research advances are needed both on the regulatory side as well as in the development of sensitive and specific analytical devices, i.e., sensors that will enable reliable food safety monitoring throughout the whole “future food” supply chain. In addition, advanced monitoring options can help in the further optimization of meat cultivation which may also reduce the currently still high costs of production.

This review presents recent advances in sensor monitoring options for the most relevant parameters in the bioprocess of meat cultivation. We provide examples of the various types of sensors and the options for their integration into bioreactors, as well as suggestions on further improvements and more advanced integration. We also briefly present the current status of the cultivated meat research and related regulation and challenges to its manufacturing, upscaling and commercialization. To the best of our knowledge, this is the first comprehensive review of the sensing options that may be of use specifically for CM production. It is our aim to highlight the need for extensive multidisciplinary research efforts in this field, focusing primarily on the sensing and related manufacturing challenges.

### 1.1. Cultured Meat (CM)

In general, cultured (or alternatively termed “cultivated”) meat production represents the production of meat without the sacrifice of animals. In other words, CM comprises products made of the cells using tissue engineering techniques [23,24,25]. There are several proposed methods for CM production, using various cell sources such as induced pluripotent stem cells (iPSCs) [26], mesenchymal stem cells (MSCs) [27] or satellite cells (SCs) i.e., the muscle stem cells [28,29]. The production of CM principally involves the generation of the skeletal muscle tissue. However, it often includes adipocytes (for fat) [30], fibroblasts, and/or chondrocytes (for connective tissues) and endothelial cells (for vascularization) [31].

The bioprocess of meat cultivation can be divided into two phases with distinct goals: **phase one** (proliferation)—with the goal to obtain the maximum number of cells from the starting batch of cells—and **phase two** (differentiation and maturation stage), where cells are seeded onto scaffolds, allowed to mature into the skeletal muscle cells and influenced into maximum protein production (hypertrophy stage). Each of these stages presents its own design requirements for the media, scaffolding and bioreactors [26,32].

Excellent reviews describing in detail different cell sources and procedures used in CM research were recently published by Post et al. [33], Zhang et al. [34], Melzener et al. [35], Bryant [36] and others.

**Figure 1:**
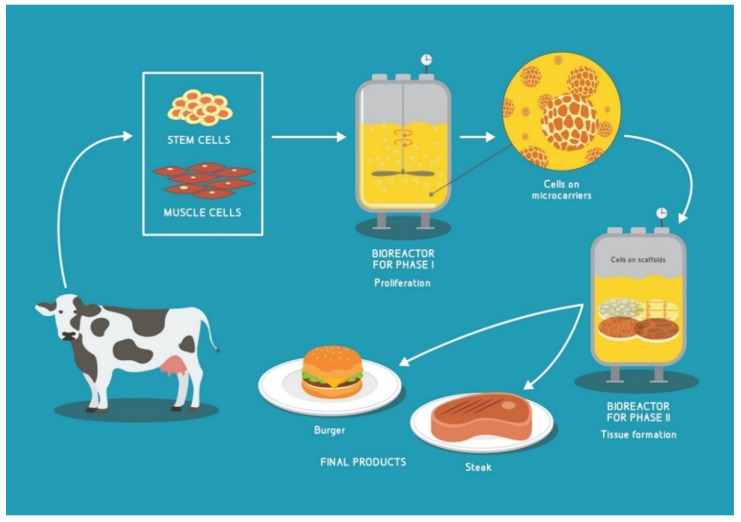
Schematics of cultured meat production.

The proposed general methodology [35,37] for the production of CA-generated meat, i.e., CM is summarized in Figure 1.

### 1.2. The Main Challenges Related to the Cultivated Meat Commercialization

The application of tissue engineering to date has principally focused on medical applications, such as regenerative medicine, whose technical principles are the same as the ones needed for producing CM. However, the difference between these two branches of tissue engineering is the much larger-scale of production necessary for CM as a product to be available as a commodity [38]. The main challenge of this large-scale production of CM is its high costs. CM production on the industrial scale is meaningful only if there is a cost-effective CM bioprocess yielding a product that tastes the same and has the same nutritional values as the existing meat products [39].

One of the most important cost-drivers in CM production is the culture medium which contains the necessary nutrients for cell growth and maturation [16,40]. The appropriate medium for commercial CM production will need to be produced free of animal-derived products (such as fetal bovine serum—FBS) and in a much more cost-efficient way than the current pharmaceutical-grade cell culture media [16].

However, besides the culture media, there are other aspects that require further innovation, including bioreactors and monitoring options, which are the main topics this review will focus on.

Another set of important challenges related to the CM commercialization is concerning the consumer acceptance of CM, which is related to the activities of the specific societal groups such as animal rights activists, vegetarians and vegans. This is an important topic whose thorough discussion is beyond the scope of this review. However, in order to better present the landscape within which the CM production and its optimization is bound to occur, we provide a brief overview of the recent literature discussing the so-called “CM community” [41,42].

The CM community was initially composed of mainly academic actors—universities, research institutes, primarily interested in the technical aspects of meat cultivation [26,32] and animal rights activists—who support the concept of CM as a more humane and animal-friendly method of meat production [43].

The next to join the CM community, coinciding with the “first lab-grown burger in 2013” [44], were the entrepreneurs and venture capitalists, who aimed to present the CM as a “transformational” innovation [45]. This brought not only new funding options to the field, but has also led to a reframing of the meaning of the term “meat” [46] and has brought into focus the importance of tissue engineering for food production [34,37,47,48,49], in the context of environmental and health issues.

The development of the CM community is strongly influenced by the notable increase in the consumers identifying as vegetarians and vegans, particularly in high-income countries and within the generation of “millennials”, as discussed in the World Economic Forum whitepaper “Meat: The Future Series” [50].

In the context of this review, it is important to emphasize the major obstacle for full CM acceptance within the abovementioned societal groups due to the use of the animal-derived components such as FBS in CM production [38,40,51,52].

The more efficient implementation of the sensors and overall optimization of the CM bioprocess may enable recycling and lower consumption of the medium and serum, while constant efforts are being made to develop a food-grade, animal-product-free medium for CM production [14,15].

### 1.3. Regulation

When comparing the current regulation frameworks of the conventional meat and of CM, it can be observed that there are certain similarities as well as notable differences. In general, the similarities are that the food safety criteria and hygiene rules need to be fulfilled for both categories, both in preparation and packaging stages (even though the packaging stage is not yet occurring for CM), as specified by the national legislative bodies. However, the CM regulation has a number of additional notions, some related to its “novel food” status and others directly stemming from the tissue engineering-based manufacturing, as discussed below.

Regulatory frameworks concerning cultivated meat differ between countries and continents. An example of this are the regulations in force in the United States (US) and the European Union (EU). In the US, federal responsibility for food safety principally lies with the Food and Drug Administration (FDA) and the US Department of Agriculture Food Safety and Inspection Service (USDA–FSIS). The FDA’s role is to regulate the manufacturing of all types of food in the US, omitting meat and poultry, with the aim to ensure its safety, nutritive values, wholesomeness and accurate labeling. On other hand, the USDA–FSIS is a service with the authority to regulate meat and poultry products under the Federal Meat Inspection Act (FMIA) [33]. In 2019, the FDA and USDA signed a formal agreement aiming to describe each agency’s role in “the oversight of human food produced using animal cell culture technology, derived from cell lines of USDA-amenable species and required to bear a USDA mark of inspection” [53].

The EU has had regulatory frameworks since the late 1990s, which differ depending on the used starting cell types. For CA-derived products, the EU mainly applies the EU Novel Foods Regulation [54] (which excludes genetically modified foods) or the genetically modified organism (GMO) legislation (which will cover the use of iPSCs for cultured meat production) depending on the used technology [38,55]. Otherwise, both regulatory systems, in the USA and in the EU, aim to assure that CM products entering the market are “safe, wholesome and unadulterated” [53].

As for the Asia Pacific region, the pre-market authorization procedure is relatively straight-forward and permissive, as shown by the Guidance on Safety Assessment of Novel Foods by the Singapore Food Agency (SFA) [56] and Food Standards Australia New Zealand (FSANZ) which plans to treat the CM under the existing standards in their Food Standards Code [31]. The world’s first regulatory approval for commercial use of a CM product has been issued in Singapore on 2 December 2020, to the San Francisco-based startup Eat Just for their cultivated chicken nugget [57].

Israel, China and Japan also appear to be moving very quickly to ensure a direct path to market for CM products [58].

## 2. Bioreactors and Scaffolding

### 2.1. Types of Bioreactors

The initial steps of the CM-process phase one (proliferation) are performed in monolayers in cell culture dishes and flasks. These steps may include the purification of isolated cells to obtain a highly purified satellite cell population which can be then kept in an undifferentiated state [29] and expanded to high numbers i.e., the desired batch amount (~10^13^). As the cell number increases, the cells are transferred to bioreactors which allow for an increased yield of cells per unit medium volume [59] through the highly controlled conditions (temperature, dissolved oxygen—DO, CO_2_, pH, mechanical stimulation) which mimic the in vivo conditions [60]. If the cells are anchorage-dependent, as a majority of the mammalian cells are, they need to be seeded onto microcarriers in order to keep them in the suspension [61]. Another option would be to grow the cells in aggregates, as shown for human mesenchymal stem cells (hMSCs) [62]. However more research is still needed to estimate the cost-efficiency of this approach for CM-relevant muscle-derived cells.

A very good review on the design of the bioreactors used in phase one for CM i.e., expansion bioreactors, was recently published by Allan, de Bank and Ellis [63]. In general, two types of bioreactors (BRs) are considered for expansion cultivation: stirred tanks and rocking platform BRs (“wave-like” [64]).

Stirred tanks are predominantly used, due to their operability and ease of manufacturing. However, the impeller-mediated mixing can induce very high shear stresses on the cells [65], which is why many CM startups turn to rocking platform BRs, that causes lower shear stress, due to gentle wave-like fluid motion in the cellbag [66]. Other promising candidates include hollow fiber [8] and air-lift [67] bioreactors, but also modifications of the conventional stirred tank and rocking platform BRs, such as by other methods for inducing the wave-motion, e.g., by using a horizontal displacement in combination with a rocking motion, which can increase the mass transfer capacity [68].

Both stirred tank and rocking platform BRs can be made as single-use bioreactors (SUBs), equipped with a disposable bag. From the economical point of view, the SUBs have several advantages, such as minimal cleaning required, fewer contamination risks due to the use of the sterile bags, and reduced downtime between batches [69]. Importantly, SUBs offer good scalability based on the already commercially available SUB configurations up to several m^3^ and versatile design options enabling a variety of mixing principles. In addition, SUBs are advantageous for small-scale parallelization [70].

Initially, the environmental impact (EI) of SUBs was thought to be their negative side, however, when the overall EI is calculated, comparing each factor, such as carbon footprint of the whole process, methods for waste disposal, as well as a full life-cycle analysis for all the materials and components, the SUBs emerge as significantly more energy-efficient when compared to the stainless steel (SS) bioreactors. The total energy consumption for the SUB system is 4156 MJ, while the SS bioreactors use almost double the amount (8018 MJ), primarily for sterilization and cleaning [71].

It is worth mentioning that prior to the use of expensive liter scale bioreactors, the bioprocess development is usually performed in spinner flasks that use impeller-driven agitation and are of considerably smaller volumes (up to 500 mL). Hanga et al. used spinner flasks for the cultivation of bovine adipose-derived stem cells (bASCs) on microcarriers [72].

For phase two—maturation, when the 3D tissue constructs (cell-laden scaffolds) are used—it is necessary to use tissue-perfusion bioreactors. Such bioreactors utilize a pumping system to perfuse the medium through the scaffold, either continuously or non-continuously [73]. Perfusion bioreactors provide a more uniform mixing of the media. This allows for better environmental control and physical stimulation of the cells in large constructs [74]. Most of the perfusion bioreactors are of similar design, comprising a pump, a reservoir for the cell culture medium, tubing circuits and cartridges, chambers or columns in which the scaffolds are placed [75,76,77]. Specifics of the design depend on the actual cell type and scaffold used. The main described types of the commercially available bioreactors are listed in Table 1. 

### 2.2. Microbioreactors

A scale-down approach and application of **microfluidic-based microbioreactors (µBRs)** are widely used due to the ability to precisely control the conditions in the cell environment and the ability to predict the laminar flow properties [86,87].

Nowadays, advanced microfluidics integrates a number of operations into a single chip, such as sample pre-treatment and preparation, DNA extraction, amplification, separation and mixing of the samples, a micromechanical system for fluid manipulation, together with optical and electronic components for signal sensing. The microfluidic technology has been widely used to study cell biology for biomedical applications [88,89], protein studies [90], pathogen detection [91,92], cell culture [93,94] or tissue engineering [95]. The dimensions of microfluidic channels and the physical scale of cells correspond to each other. Thanks to this, it is possible to properly monitor and manage different cellular microenvironment parameters of the cell cultures [96].

Scientists are increasingly focusing on developing 3D cell cultures that would better replicate the in vivo conditions of an organism [97,98]. These cultures allow cells to grow in multiple directions, which is a step-up from the planar surface of the 2D cell cultures [99]. Furthermore, it has been observed that the response and function of 3D cell cultures are greatly enhanced under the flow conditions, i.e., in perfusion systems [94,100].

µBRs are one type of the instruments that can be used for providing flow conditions for 3D cell cultures in a very cost-efficient way. Incorporating 3D matrices into microfluidic platforms will combine the specific advantages of these two systems. Microfluidics require the use of only small volumes and dilution is kept to a minimum, while 3D matrices allow cell cultures to behave as if they were in vivo. Signaling and various other phenomena such as flow-induced stress on adherent cells [101] could be directly analyzed within µBRs and generated experimental data can be used for benchmarking the mathematical and computer fluid dynamic (CFD) models, as shown in Section 3. In addition, there is a growing body of research concerning the integration of the scaffolding materials in microfluidic 3D cell culture systems [102].

A particularly interesting application of the microfluidics is in **scale-down analysis**, where compact microfluidic platforms integrated with diverse sensing technologies have been used for the analysis of different bioprocessing to resolve scale-up problems, since the microfluidic devices enable a significant reduction in time and cost of bioprocess development, and allow a high degree of process parameters control, subsequent treatment and analysis [103,104]. Simply put, the evaluation of different sensor prototypes is more efficient in such a controlled system as µBRs [105].

It should be mentioned that standard cell culture techniques cannot be directly transferred to microfluidic environments without consideration of the physics of the microscale, primarily laminar flow, reduced transport times of mass and heat, etc. [106].

Although a different topology of the microfluidic chip has been used for cell culturing, some design considerations are mandatory for microfluidic cell cultures, such as the selection of an appropriate material for the microfluidic chip, the dimensions and geometry of microfluidic bioreactor, and setup of the fluid flow.

Different technologies were used for the fabrication of microfluidic microbioreactors, but the PDMS (polydimethylsiloxane) is widely used [107]. PDMS polymer has many advantages for the fabrication of µBRs such as biocompatibility, optical transparency, and mechanical flexibility. The main drawback of the PDMS process is a chip fabrication complexity that relies on a non-trivial lithography method and, for the chip re-design, it is necessary to repeat the complete fabrication flow. The alternative to the PDMS in terms of biocompatibility is glass. Glass has advantages over other materials in terms of optical transparency, good insulating properties and surface stability, and mechanical and temperature resistance. However, the fabrication of precise microchannel on glass for microfluidic chips is still challenging and additional insulation layers are required for bonding between different layers [108].

µBRs can also be fabricated in ceramic-based low-temperature co-fired ceramic (LTCC) technology, thanks to the possibility to create complex multilayered structures [109]. LTCC-based microfluidic chips have good chemical and temperature stability and very good mechanical properties. Unfortunately, there are drawbacks to LTCC technology, such as complex fabrication that requires a clean room facility as well as the non-transparency of the LTCC material. Thus, it is necessary to perform bonding of the LTCC structure with other transparent materials (such as PDMS or glass) for visual control of the process.

The next interesting technique for microfluidic device manufacturing is the 3D printing process, through applying additive manufacturing. 3D printing allows the creation of complex shapes, quickly and in a cost-effective manner based on thermoplastic filaments, such as acrylonitrile butadiene styrene (ABS) and polylactic acid (PLA) [110]. The structure is created layer-by-layer, and the final 3D structure is distortion- and delamination-free. However, the limitations of this process are the low resolution of the fabricated channels, and an assortment of materials, which are usually not optically transparent.

A xurography can be used as a rapid prototyping technique for the rapid manufacturing of low-cost microfluidic devices, since it does not require expensive clean-room facilities [111]. A good resolution of the channels can be obtained with precise plotter cutting in polyvinyl chloride (PVC) foils. PVC is also a material that has good biocompatible characteristics for biomedical applications and good optical transparency, but its melting temperature is relatively low and therefore it is not suitable for sterilization in the autoclave. Similar problems exist with other thermoplastics. Recently, a novel thermoplastic elastomer material—Flexdym—is being advertised as a cost-effective alternative to PDMS technology [112,113]. The proposed technology enables rapid fabrication by thermal molding and fabrication of microfluidic chips in 30 s. Besides rapid fabrication, the proposed technology enables low resolution of fabricated channels, up to 50 nm. Flexdym is characterized by good optical transparency and biocompatibility and therefore presents a promising material for different future µBR applications.

Another polymer that enables rapid manufacturing of microfluidic channels with a wide range of applications is poly(methyl methacrylate)—PMMA [114,115]. PMMA has better mechanical properties than PDMS and is more robust and can be processed easier than traditional materials such as silicon or glass. The fabrication of precise microchannels can be achieved by CO_2_ laser cutting, graving, or micro-milling techniques. The multilayer structure requires additional thermal bonding between layers and the advantage of PMMA is that it can be bonded with a number of different substrates, including glass, silicon and PDMS [116].

Recently, different hybrid technologies that combine different materials or alternative fabrication processes were proposed to overcome the above-mentioned drawbacks of precise channel fabrication and multilayer bonding [117,118]. The selection of the appropriate microfluidic chip fabrication technology and materials depends on the application, chip complexity, operating temperature, required optical properties and many other factors.

### 2.3. Microcarriers and Scaffolds

Scalability is one of the main challenges CM research needs to address. In this context, it was necessary to develop techniques that allow for the efficient culturing of the anchorage-dependent mammalian cells which are the main constituent of cultured meat. Such techniques include aggregate cell cultures, fixed bed reactor cultures, and microcarrier (MC) cultures, with the latter being the most promising due to their high surface-to-volume ratio [119]. Many MCs are developed and commercially available for cell lines typically used in the medical field, which is why experiments with cells on microcarriers were mostly carried out with human mesenchymal stem cells (hMSCs), but it is shown that bovine myoblast cells have similar behavior in vitro [61]. MCs are also convenient for the culture of other types of anchor-dependent cells such as insect, fish, and avian cells [120]. MCs are also relatively affordable and can be easily implemented in various bioreactors [121].

Microcarriers possess properties suited for different types of cultured meat production. They can serve as acting substrates to which cells can attach and proliferate. However, such MCs need to be either dissolved/degraded in the early stages of the process or separated from the cells at a later stage. MCs can also be incorporated into the final product if they are composed of edible materials [119]. Edible polymers that can be used for MC production include polysaccharides (e.g., starch, chitosan, alginate, and others of plant and animal origin), lipids such as shellac and paraffin, polypeptides such as collagen, gelatin, and pectin, and synthetic, inert polymers such as polyethylene glycol (PEG) and polyglycolic acid (PGA) [122].

Most of the early MCs were in fact based on synthetic polymers such as PLGA, polyhydroxy-ethyl-methacrylate, acrylamide and others [123]. Such microcarriers can easily be manufactured in large quantities, however, they are usually lacking recognition sites for cells. This limits their application in the expansion of cells [124]. Natural polymers and materials derived from them are recently being targeted for research as they are easily obtainable and can be biocompatible [125]. Some of the most common natural polymers, such as cellulose, alginate, and chitosan, are viable candidates for use in the upscaling of cell expansion, due to their biocompatibility and biodegradability [126,127]. However, there are still MC-related issues such as seeding density, efficiency of cell attachment to the MCs, bead-to-bead transfer and efficiency of cell harvest from the beads that require further extensive research and optimization [128,129].

An ideal alternative to MC-based myoblast culture would be a single cell suspension culture. Additionally, using different biochemical modifications the adherent-prone cells could be made anchor-independent. These suspension cultures are being targeted by novel methods, however, still on a smaller scale [130].

The type of scaffolding material needed for the full in vitro muscle tissue formation process (phase two) is a three-dimensional (3D) scaffold that mimics the in vivo environment of living cells—extracellular matrix (ECM). This material provides mechanical support and can even enable the potential vascularization of the tissue construct. As such, scaffolding represents one of the key components of cellular agriculture. Scaffolding materials provide a large surface for cell attachment and growth and an integrated network that supports cell expansion and differentiation in an anchor-dependent manner. This porous network maximizes medium diffusion, allowing the flow of oxygen and nutrients, as well as the removal of waste, in order to maintain cell metabolic functions and avoid necrosis.

When it comes to the use of scaffolding in cellular agriculture and food production, there is a specific set of criteria that must be met. The final product contains the scaffold as one of its main components, therefore the scaffold should be degradable or easily dissociated from the tissue without leaving behind material traces [33,131]. For consumption, scaffolding biomaterials should have a specific texture, thermal stability, certain nutritional values, and be safe to eat (non-toxic and non-allergenic) and tasty, be it cooked or uncooked [37,132].

Keeping in mind one of the key aspects of CA, which is the humane treatment of animals, one should stay away from e.g., animal-derived collagen, gelatin (hydrolyzed collagen) and similar livestock products when considering different scaffold options. These materials do not self-replicate and large quantities of livestock are needed as a source [33]. Materials that show more promise for use in CA are polysaccharides such as starch, structural fractions of cellulose (amylose and amylopectin), chitin and chitosan of fungus, alginates, hyaluronic acid, pullulan and others [132,133,134]. However, some of them may pose the risk of allergies e.g., alginate products [131]. A textured soy protein was demonstrated by the Levenberg group at Technion Institute of Technology, Israel to function well as a CM scaffold, leading to the formation of a 3D engineered bovine muscle tissue [132] and the “world’s first cultivated steak by Technion-related company Aleph farms” [135].

Through recombinant technology, scaffolding based on proteins can be made to incorporate different materials such as silk, keratin or fibrin. Naturally-occurring polyesters (polyhydroxyalkanoates), produced by bacteria are also of particular interest in this regard [136]. Apart from natural, a number of synthetic polymers can be considered. In general, these systems are safe for human consumption and can be designed to have a customized rate of degradation achieved through chemical hydrolysis [137]. Systems based on synthetic polymers are of consistent quality and supply, however they can be limited by production costs and the necessity for surface functionalization.

Lastly, composite matrices of plant and microbial origin, such as lignins, decellularized leaves and fungal mycelia, are also actively being pursued [138,139]. For example, decellularized apple hypanthium has been demonstrated as a 3D cell culture substrate. This type of scaffold allowed Henrietta Lacks (HeLa) cells, 3T3 fibroblasts and C2C12 murine myoblasts to proliferate for up to 12 weeks [138].

Some of the newest alternatives are presented in the pre-print server-published work by Holmes et al. describing bread-derived scaffolds in the form of a highly porous crumb (the soft, inner part of the bread) [140]. Using this type of scaffold, multiple cell types relevant to the development of novel future foods can proliferate in 3D. This yeast-free type of bread scaffold (“soda bread”) was able to maintain its mechanical stability over two weeks in culture conditions. Importantly, bread-derived scaffolds are cost-efficient and convenient for scale-up [140].

Meat from livestock is composed of muscle, adipose and connective tissue [25]. The formation of such complex tissue construct has to be properly coerced by the properties of the scaffolds. However, in order for the scaffold to be suitable for both muscle and adipose tissue formation, it needs to have appropriate stiffness for both tissue types, which is not a trivial task to fulfill, since muscle tissue needs a much more rigid and stiff scaffolding than the adipose tissue does [141,142]. This is why it is still challenging to design one solution for all types of meat components [30].

### 2.4. 3D Bioprinting for Cultured Meat 

One way to generate 3D cell-scaffold constructs is to use 3D bioprinting technology, i.e., additive biofabrication [143,144,145]. A suitable biomaterial can be printed simultaneously, and will serve as the scaffold for the printed cells [146]. However, to 3D print muscle analogs characterized by high cell alignment and synchronous contraction, some critical barriers have to be understood and overcome. These include resolution, throughput, chemical and biological compatibility [147] as well as the effects that the biofabrication process can exert on myoblast growth. Distler et al. show that the appropriate selection of the bioprinter’s nozzle size and extrusion pressure enabled them to achieve guidance for the mouse myoblast cells (C2C12) on the oxidized alginate–gelatin (ADA-GEL) hydrogel matrix. The exerted shear stress was guiding the cells in the direction of the printing, in which they continued to grow and differentiate into ordered myotubes [148]. The mechanical properties of “meat-ink” are also one of the factors affecting the printability of meat products [149].

The benefits of 3D bioprinting of cultured meat are multiple, such as precise regulation of protein, fat, and other nutritional content [150], speed of production, ability to produce relevant forms i.e., steak-like form which can lead to greater consumer acceptance [151], and adaptation for use in extreme conditions such as space [152]. The world’s first cultivated ribeye steak has been 3D bioprinted by the Israeli company Aleph farms in February 2021 [153].

## 3. Mathematical Modeling and Computer Fluid Dynamics (CFDs)

Mathematical modeling is a powerful tool that enables better understanding and the prediction of complex bioprocesses, and thus allows for the optimization of different output variables and processes with the purpose to predict the most efficient properties for process control. In complex systems such as bioreactors (BRs), different biochemical processes during cell growth are occurring in parallel with fluid motion such as oxygen and carbon dioxide transport, heat transfer, mass transport, cell division and growth process, etc. However, the coupled models of hydrodynamics and cellular systems are rare in the literature, due to the lack of suitable software that couples the physical and cell culturing processes. Therefore, a lot of computational models are focused on the optimization of fluid behavior in bioreactors, while a lot of biological models are focused on the cell culturing process in the bioreactors. Hence, considering the complexity of a bioreactor system, and the importance of controlling and predicting systems’ behavior, different predictive computational models were proposed in the literature based on computational fluid dynamics (CFDs) [154,155,156,157,158,159,160,161].

The **microbioreactors (µBRs)** are relatively simple for modeling, due to the predictability and repeatability of fluid behavior, and initial parameters such as temperature, pH, amount of oxygen, and carbon dioxide in the system. There is a number of computational models for µBRs available in the literature, with a particular focus on the analyses and optimization of fluid behavior and mass transfer in the system, proposing efficient mixing methods for the µBR system [155], flow field and oxygen transport [156,157], mass transfer, fluid pressure and shear stress [158] and enzyme adsorption [159].

Contrary to the laminar flow in µBRs, which occurs when the liquid moves in parallel layers with minimal lateral mixing, the turbulent flow, typical for the BR systems with mixing, is characterized by chaotic variations of velocity in space and time. With the systems’ scaling up, the system is becoming more complex and additional processes have to be taken into consideration. Besides the prediction of the turbulent flow behavior, the complete model of the BR has to also consider the gas exchange in the system, heat transfer, shear stress, mixing efficiency and foaming of the medium. Different models of BRs were recently proposed: single-phase ones regarding only the liquid in BR [162,163], two-phase models including cells [164,165] and three-phase models including additional gases in the BRs [166], reviewed in the following subsections.

### 3.1. Modeling for Stirred Tank Bioreactor

The mixing process in the stirred tank BR systems is an important part of modeling and testing BRs, due to the appearance of “dead zones”. In such dead zones of the BR, the impellers that rotate in the tank do not make turbulence, hence this “dead zone” part of the BR is not involved in overall agitation. In the dead zone, a slow process of diffusion is taking place, which causes the whole system to be inhomogeneous.

In order to optimize the mixing process, recently proposed studies were examining an optimal impeller configuration for stirred tank BRs by using CFD [160,161,162,163,164]. The proposed geometries of impellers were based on Segment–Segment, Segment–Rushton [160], Scaba, Paddle [161], Elephant ear [162], blade turbines [163], and radial geometry of impellers [164]. The impeller shape has an important influence on cell cultivation due to the stress it causes. CFD enables one to examine the optimal speed of rotation of the impeller as well as calculations of velocity profile and gradient, and different parameters such as flow number and mixing time.

Different models for stirred tank BRs were recently proposed in the literature. The simplest model considers only liquid phase in the system, and the analyses performed in these models are related to turbulent flow properties and kinetic energy of the flow [163]. More advanced studies involve the second phase into the model—biomass i.e., the cells. This is enabled by coupling CFD with Monod kinetic equations [165], equations that describe the cell growth in the stirred tank BR. Additionally, the proposed model shows a good agreement with glucose concentration, biomass, DO, gluconic acid [166].

The recently proposed research couples CFD with population balance equations (PBE) in order to examine biomass production in parallel with turbulent flow characterization. The influence of three different impeller models was examined for mixing efficiency and the results have shown the Scaba design to have the best properties [161].

The integration of different sensors in the BR enables the online monitoring of different parameters, important for cell cultivation. The main challenge in the turbulent BR systems is to find an optimal place for sensor positioning. The sensor integration enables the automatization of the cell culturing process without the need to take a sample and perform offline measurements of their improvement. On the other hand, considering the changeable behavior of turbulent fluid motion, for reliable results it is important to find an optimal place for the specific sensors. A study by Rudniak et al. examined the optimal position for the temperature sensor in the stirred tank chemical reactor [167]. Examination of an optimal sensor position was carried out in CFD simulations by simulating exothermic reactions in homogenous and heterogenous systems and compared with the experimental results from calorimetric measurements. Therefore, an adequate mathematical model in combination with empirical benchmarking can significantly aid in the sensor system integration and allow for the better understanding of different parameters inside the BR, resulting in an improved cultivation process.

### 3.2. Modeling for Rocking/Wave Bioreactor

Rocking/wave BRs use wave-like movement for the mixing of the cell culture medium, shown to be a very suitable solution for culturing cells that are sensitive to shear stress, such as the majority of anchorage-dependent cells [66]. Important specific parameters for the wave BR modeling are the angle and speed of the rocking motion, as well as the standard ones such as mass transfer, oxygen transfer rate, flow profiles and shear stress.

A comparison of different operating conditions for wave BR model with a volume of 10 L was reported by Zhan and coworkers [168]. The influence of parameters such as the angle and rocking speed were examined and the results were explained by assuming that the resonance phenomena can occur in the wave BR. The CFD simulations showed that increasing the rocking angle can increase the shear stress and mixing in the BR. However, they also show that the increasing rocking speeds do not directly increase the mixing and the shear force, which is explained by a resonance phenomenon. The resonance caused the lowest studied rocking speed, 15 rpm, to generate the highest fluid velocity, mixing and importantly, the highest shear stress compared to the higher speeds of 22 and 30 rpm [168]. These findings need to be taken into consideration for bioprocess optimization for shear-stress sensitive cells.

Different studies examined oxygen mass transfer in wave BRs and oxygen transfer from headspace gas into the liquid [169,170,171,172,173,174,175]. A recent improvement in the field of understanding of oxygen mass transfer was made by a mechanistic model of Bai et al. [176]. The analyses have shown that the complete contribution of oxygen transfer is made of two independent mechanisms: wave breaking air entrainment made by the wave-like motion of BR and surface aeration. At lower rocking frequencies and angles, the surface aeration was dominant, while the rocking frequency and angle increase the contribution of wave turbulence and the complete mass transfer also increases.

## 4. Sensors

The application of sensor systems for monitoring the cultured meat production can be extremely beneficial at the production scale, since they may allow better in-process control and re-optimization of the culturing process, saving on medium usage and providing overall cost reduction for the whole bioprocess.

**Figure 2:**
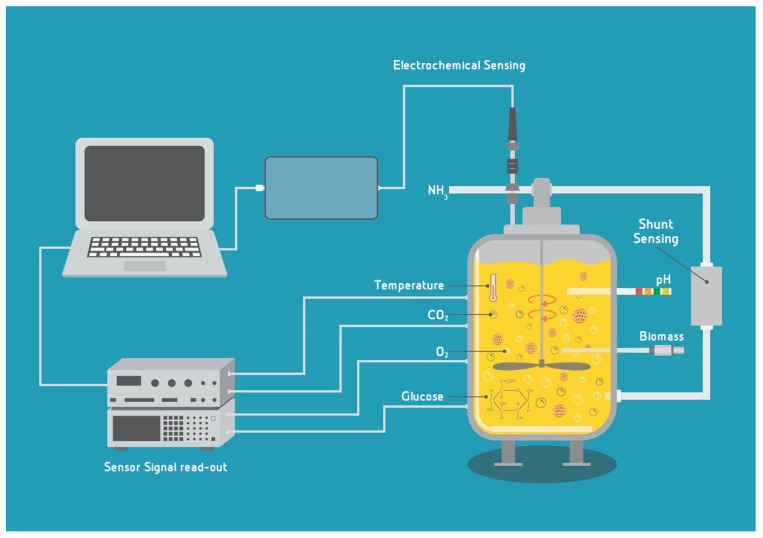
Main milieu parameters in meat cultivation.

A very good categorization of the relevant parameters to be monitored for the engineered tissue in a bioreactor was made by Starly and Choubey [177] and Wendt et al. [178] comprising the milieu parameters and the construct parameters. The milieu parameters are the physical ones (temperature, pressure, flow rate, viscosity, etc.), chemical (pH, dissolved oxygen—DO—and CO_2_, volatile gasses), and biological and chemical parameters (growth rate, biomass, cell morphology, viability, concentration of the nutrients and metabolites) of the cells and medium (Figure 2).

The sensing options can be in general further divided into (a) **invasive (embedded) or in-line sensors**—where the sensing probes are immersed in the culture fluid or directly contact the tissue construct; (b) **non-invasive (non-contact) sensors** that are placed outside of the bioreactor chamber and perform monitoring via, e.g., spectrophotometry or ultrasound and (c) **indirect (at-line) sensing**—performed on the culture medium, via sampling, either as **off-line** (“quasi on-line” [179]) analyses or shunt sensing [178,179].

The requirements for each category are different since, e.g., in-line sensors need to be sturdy enough to undergo the sterilization process and the temperature kept inside the bioreactor, while the off-line sampling will require manual work and sample manipulations, which are both prone to errors and possible contaminations. At-line sensing can be performed via a shunting loop and the analyzed media can be either returned to the bioreactor chamber (proper sterility of the shunt needs to be maintained) or discarded. The sensors for indirect sensing can be more advanced, specific and sensitive than the inline ones, since they are not being placed into harsh conditions of the inside of the bioreactor.

Therefore, the construction of the sensors, their operating principle, and accuracy depend on the type of reactor in which they will be implemented and on their position. An ideal sensing option would be the one that is automated, measures in real-time and on-line (continually) and is responsive, i.e., is connected to the whole cultivation system and has a feedback loop mechanism to the culture regulation. The new sensing options are needed not only in order to provide better bioprocess control, but also to potentially enable media recycling (in combination with filtering), which may reduce overall costs of CM cultivation, since the medium is one of the major cost-drivers of the CM bioprocess. In order to develop new sensing options specifically for CM, it is useful to combine mathematical modeling and computational fluid dynamics (CFD) coupled to experimental validation to develop and optimize sensors and enable their integration into commercial or newly designed bioreactors.

### 4.1. Sensing Options for pH, DO, CO_2_ and Temperature

Standard bioreactor monitoring includes the measurement of physical variables such as temperature and pressure in combination with several chemical parameters of the culture such as pH, dissolved oxygen—DO, and concentration of CO_2_ [180,181,182,183,184].

The **temperature** inside the bioreactor chamber is crucial to ensure optimal cell viability and product growth rate. The optimal temperature depends on the type of cells used [185]. The culture of mammalian cells regularly happens at 37 °C, while the fish cell culture is maintained in the temperature range between 15–30 °C [186]. Therefore, the process temperature control in the range of 10–40 °C with an accuracy lower than 0.5 °C is required to avoid loss in production. A number of different methods can be used for temperature measurement inside or outside of the bioreactor [187]. Thermocouples, devices composed of two dissimilar electrical conductors, are the most used temperature sensors due to their relatively low price. However, thermocouples also have low sensitivity of the sensor. On the other hand, the resistance sensor measures the temperature based on the electrical resistance change in metal wire usually made of platinum, zinc, nickel, or copper. Resistance sensors are also widely used due to their high accuracy and faster response time. A number of resistance sensors and thermocouples exist on the market with different accuracy and operating ranges specifically designed to be implemented inside different types of bioreactors [188,189,190,191,192]. Other temperature sensors such as the ones based on thermistors (sensitive resistor made of metal oxide), gas/liquid-filled thermometers (where the volume of fluid changes with the temperature), bimetal (composed of two metal strips with different thermal expansion coefficients), silicon bandgap temperature sensors (where temperature depend on the forward voltage of a silicon diode) or infra-red (IR) sensors (which infers temperature from a portion of the thermal radiation) [193,194] may be used, however, these are not recommended for the highly accurate bioprocesses. Nevertheless, they might be a good choice for the single-use bioreactors, since some are relatively cheap and can be integrated with the SUBs. Complementary metal oxide semiconductors (CMOSs) and thermistors can be used for non-contact measurement from the outside (IR sensors). Multipoint temperature measurement systems with several temperature sensors integrated at different positions are used for the industrial-scale bioreactors, since they can provide better culture control and ensure stable system operation [190]. Examples of the commercially available temperature sensors are shown in Table 2.

The **pH** of the cell culture medium can provide information about cell growth rate and metabolism since the lower pH indicates buildup of the acidic waste products (such as lactates and carbonic acid). The optimal pH for animal cell culture is ~7.4. Even a small change of 0.1 pH units from the optimum can have an extreme impact on cell viability and growth rate. In bioreactors, pH is typically monitored using electrochemical and optical sensors [187,195]. The electrochemical pH sensor is composed of an ion-selective silver or silver chloride working electrode housed in a glass selective membrane and immersed in a chloride solution. The working electrode measures the change of the potential between the internal solution and the analyte across the membrane in comparison with the potential of the referent silver electrode. The referent electrode is enclosed in a plastic or glass tube filled with an understood electrolyte such as KCl, and it is separate from the analyte. The main drawback of the electrochemical electrodes is their bulky size and fragile construction. On the other hand, the optical pH sensor is characterized by much smaller dimensions and simpler construction. Optical pH sensors measure the optical absorbance or fluorescence of a pH indicator dye bound to the sensor surface [187,195]. Typical indicators used in pH optical sensors are various pH-sensitive dyes such as cresol red, phenol red, bromophenol blue, or 8-hydroxy-1,3,6-pyrene trisulfonic acid. Indicator dye is immobilized (coated) onto a solid substance, usually composed of synthetic polymers. Different combinations of dyes and immobilization processes have been proposed in the literature to extend the measurement range and accuracy [194]. The main disadvantages of optical-based pH sensors are cross-sensitivity to, e.g., ionic strength and temperature and their limited dynamic range. Therefore, the calibration of the optical sensor needs to be performed before the integration inside the bioreactor and often an additional recalibration is required during the culture process. This is time-consuming and can lead to contamination. Another drawback of the optical sensors is the slow response time (a range of a couple of minutes), which can be significantly improved by the application of the luminescence-based pH-sensitive coating hydrogels directly on the optical fibers and optimization of the coating thickness [196]. A number of the above-mentioned types of pH sensors are available on the market with different measurement ranges, response times, and constructions [191,197,198,199,200]—Table 3. In conclusion, it can be said that the small size and good sensitivity of the optical pH sensors make them suitable for implementation in the small-scale culture systems, while the electrochemical sensors are still the most used in the larger, industry-scale systems.

Another important parameter that requires constant monitoring in cell cultivation is **dissolved oxygen (DO)** which has to be continually delivered in order to meet cellular metabolic demands and to avoid reduction in cell growth and viability. Specific cell lines have different oxygen utilization rates and therefore have different oxygen requirements. The dissolved oxygen in bioreactors is usually measured using electrochemical, optical or paramagnetic sensors.

The standard electrochemical DO sensor, known as the Clark-type sensor, is composed of an anode and a cathode, both placed in an electrolyte solution, and an oxygen-porous membrane used to casing the cathode [179]. Dissolved oxygen molecules diffuse through the membrane and are reduced at the cathode when the cathode is polarized with a constant voltage. This reaction results in a current flow proportional to the concentration of the DO in the solution.

Most current electrochemical DO sensors consist of a zinc or lead anode and a gold or silver cathode placed in an electrolyte solution. They use two types of metal for the electrodes and their different reaction with electrolyte results in an electromotive voltage proportional to dissolved oxygen [201]. Electrochemical DO sensors are characterized by good compactness, but low response time and short lifetime due to the degradation of the porous membrane.

Optical DO sensors use an optical system to measure oxygen based on the photoluminescence quenching by the oxygen-sensitive indicator [179,193]. The oxygen-permeable polymer matrices immobilized with complexes of ruthenium, palladium or platinum are used as a sensitive layer in the oxygen-sensitive indicators. When these molecules are irradiated with an excitation beam, they have red luminescence. When molecular oxygen is present, the photoluminescence of such molecules is quenched by the mechanisms which are still not fully understood [202], leading to a decrease in red luminescence. Hence, the duration and intensity of the red luminescence are inversely proportional to the concentration of oxygen molecules.

Optical sensors have a long shelf life compared to their electrochemical counterparts, but a slower response time. Additionally, electrochemical sensors generally perform best at higher concentrations of oxygen whereas optical sensors are suitable for lower oxygen levels.

The paramagnetic sensors’ operating principle relies on the fact that oxygen is a paramagnetic gas that is attracted to a strong magnetic field. Importantly, in the vast majority of bioprocesses, oxygen is the only paramagnetic gas present in the bioreactor, which makes this type of sensing highly selective [179,201]. The paramagnetic sensor is usually composed of two nitrogen-filled glass spheres. When the sensor is placed in the strong magnetic field, the oxygen in the surrounding fluid is attracted to the magnetic field, resulting in a force on the spheres that are mounted on a rotating suspension. The DO in the fluid is proportional to the strength of the torque acting on the suspension [203]. Paramagnetic sensors are applicable for DO variations from 0% to 100%.

A number of DO sensors exist on the market specifically designed to be implemented within bioreactors—Table 4. However, the optical ones are still the most used due to their price, lifetime, and accuracy [179,204].

**Carbon dioxide** in a cultivated meat bioprocess is closely linked to the cell density, and can readily diffuse across the cell membrane, affecting cellular metabolism and resulting in a lower intracellular pH. The determination of dissolved CO_2_ is more difficult due to its chemical reactions with water and the cell culture medium buffered with the CO_2_-bicarbonate based buffer [206].

In the bioreactors, CO_2_ is usually measured using an electrochemical sensor based on the Severinghaus electrode. This type of sensor uses an optical system to measure the CO_2_ partial pressure indirectly by measuring the pH value changes in the bicarbonate solution [207]. The pH indicator is separated from the analyte solution by a CO_2_-selective membrane made of polytetrafluoroethylene or silicon. This pH value is dependent on the amount of carbon dioxide reversibly flooding through the membrane into the electrolyte. The concentration of CO_2_ is measured using luminescent or colorimetric principles. 

Mills [208] summarized the different optical sensors for the detection and quantitative analysis of carbon dioxide. In general, the diffusion of CO_2_ through the selective membrane is a relatively slow process, which is why a carbon dioxide sensor has a slow response time. CO_2_ sensors suffer from low-temperature stability and therefore require additional temperature compensation. The Severinghaus CO_2_ sensor remains accurate at 0–30% CO_2_ and loses accuracy with higher concentration. Unfortunately, the shelf-life of the selective membrane is an additional problem, causing CO₂ sensors to require periodic maintenance in terms of membrane replacement and recalibration. 

Nowadays, infrared (IR), non-dispersive infrared (NDIR), acoustic methods, conductometric sensors, and thermal conductivity measurements are also used for the determination of CO_2_ [209]. Unfortunately, most of these sensors are not applicable for integration within the bioreactors. Therefore, the main research efforts related to the CO_2_ sensors are directed towards developing sterilizable resistant sensors, extending their measuring range, as well as their service life and calibration intervals. These efforts led to the development of solid electrolyte CO_2_ sensors with short response times for the in situ measurement, and miniaturization and improvement of selectivity and sensitivity of IR sensors [209,210]—Table 5.

Considering that O_2_, CO_2_, pH and temperature are the crucial parameters for all cell culture processes, the recent trend in the sensor development for CM cultivation is directed towards the integration of two or more above-mentioned sensors inside one automatic acquisition module that can be easily mounted or integrated inside the bioreactor [197,205,212].

### 4.2. Biomass Sensors in Bioreactors

Biomass describes the progress of cell growth in the BR during cultivation. Therefore, it is one of the most important parameters for monitoring over time. Besides the cell concentration progress, it is important to measure the viability of the cells in the BRs. A recently published review by Busse et al. refers to different approaches for biomass estimation and summarizes all the biomass sensors available for SUBs [193]. Some of the proposed direct methods include manual cell counting, near-infrared (NIR) spectroscopy [213], and dielectric spectrometry [214]. Indirect proposed methods are based on measuring gases released during the bioprocess [179,215], glucose uptake [216], and redox potential measurements [217]. Many of the proposed techniques have their drawbacks, and the proper solution for integration is still one of the most challenging topics in the field of biomass sensors.

Noninvasive and nondestructive spectroscopic methods are widely used for biomass estimation [195,218,219]. Recently proposed optical sensors for the biomass detection principle are based on relations between biomass and different chemical processes during cell growth. A modified commercially available optical sensor [220] is used for biomass estimation based on the relation between biomass and lactic acid production that can be measured by scattered light in the infrared region. The proposed sensor enables online measurements and a linear dependence between the optical signal and biomass concentration and shows the ability to measure the viability of the cell culture system [221]. In addition, recent progress was made in the multifunctional platform for measuring biomass, pH, and O_2_ in single-use shake flasks [222]. In general, the problem with the detection of optical sensors in BRs is the uncertainty of cell contribution to the signal. Concretely, the final signal consists of cell contribution, and some irrelevant contributors such as non-cellular solid particles, and bubbles in the system. Additionally, the limitations of optical detections are related to the inability to adapt to cell morphology changes due to the growth process or aggregation, which can be misinterpreted [215].

The impedimetric principle is estimated as a high potential principle for the commercial use of sensors. Radio frequencies (RFs) are usually used for biomass estimation in BRs because in this frequency range the dielectric specter includes β dispersion. Concretely, cells behave like small dipoles in the RF frequency range due to the charged ions that collect at the opposite sides of the cell membranes in the AC current field. Consequently, the number of cells directly influences the dielectric permittivity as well as the capacitance of measured impedance. A detailed overview of the dielectric spectroscopy principle is described in the publications by Carvell et al. [223]; and Markx et al. [224].

The impedimetric approach has shown large potential for in situ measurements for CM production due to the possibility for the estimation of biomass in cell suspension as well as in the medium with the cells seeded onto the microcarriers. Recent studies with dielectric spectroscopy measurements [217,223,225,226] use commercially available probes for capacitance measurements [221,227,228,229].

Different solutions for improving biomass sensing are proposed. For example, a low-cost sensor for biomass measurements in single-use bioreactors (SUBs) based on coplanar transmission lines had shown a good correlation between optical density and effective permittivity at a frequency of 1 kHz [230]. Finally, one recent study was examining the scalability and transferability of the commercially available capacitive sensor (BioPAT^®^ ViaMass, Göttingen, Germany) and its integration in SUBs. It is shown that the proposed sensor can be integrated into SUBs from 50 to 2000 L reactor volume. However, the authors also state that the capacitive sensor can estimate the total number of cells, but not their viability [227].

Several capacitive sensors for biomass and viability measurements are commercially available on the market. The sensors can be connected to the BRs during measurements and enable the real-time monitoring of cell culture progress. The sensors’ main properties and types of BRs these sensors are intended for are listed in Table 6. The sensors use the frequency range in RF. As it was mentioned, this property enables the detection of different concentrations and cell viability.

All of the sensors listed in Table 6 are suitable for bacterial and animal cell culturing, while one of them, a sensor by Sartorius [231] also supports plant cell cultivation. Due to the influence of cell morphology and size on the measured results, different sensors have different resolutions. Consequently, the smaller cell size orders larger resolution, so the bacterial cells with the size around 1 µm will have the highest resolution, compared to the animal cells with a size range of tens of microns and finally, the lowest resolution will have the largest plant cells with the size of up to 100 microns. The proposed sensors are customized mostly for bench scale BRs, such as the sensor Standard Remote Futura [232] used for volumes up to 100 mL. However, some (such as BioPAT^®^ ViaMass [231]) are applicable for volumes up to 100 L [233].

### 4.3. Electrochemical Biosensors for Nutrients and Metabolites

Cell growth is associated with the consumption of the carbon source, amino acids, vitamins and other essential nutrients and the production of byproducts (metabolites) such as lactate and ammonia. While the intrinsic effect of lactate on cell growth and productivity is a matter of debate, it has long been known and confirmed by numerous studies that increased ammonia (NH_3_) levels are toxic and inhibitory for mammalian cell cultures [235,236].

In addition, the monitoring of levels of nutrients such as glucose and amino acids is important for overall bioprocess control and can be particularly important for enabling efficient medium recycling. One can conclude that having sensitive sensors for both the nutrients’ and metabolites’ concentrations in combination with filtering would allow for potential media recycling and significant cost reduction of the CM bioprocess.

**Electrochemical biosensors (ECBs)** are particularly useful for the quantitative analysis of cell culture nutrients and metabolites. ECBs contain a transduction element, frequently covered with a chemical or biological recognition layer for enhancing sensitivity and selectivity, which interacts with the target analyte and produces an electrical signal. The signal is proportional to the analyte concentration either linearly (voltammetry, amperometry, conductometry) or logarithmically (potentiometry). ECBs can be also efficiently miniaturized for the detection of different metabolic parameters in cell culture media and nutrients determination [237,238].

Among the ECB sensors for nutrients and metabolites, **glucose sensors** have been particularly developed, which is mostly due to its medical importance in diabetes treatment. A number of glucose sensors have been developed for this purpose, and many of them are commercially available on the market [239,240,241,242]. From the point of view of the cell culture, glucose is one of the most important nutrients which cells consume in the bioreactors (BRs). During the cell growth in BRs, the real-time measurement of glucose levels has to be performed in order to control and understand the cell metabolism. Therefore, the glucose sensors intended for BRs have to work continuously over time and enable online measurements while the cell culture process occurs. Besides direct sensors, different other methods for glucose levels monitoring in the cell culture are proposed, based on Raman spectroscopy and liquid chromatography [243,244,245,246]. Although most of the proposed solutions show good sensitivity, most of these solutions, even the commercial ones, are not adapted for integration within BRs and application in cell culture for continuous monitoring.

Different principles of glucose sensors based on electrochemical and optical detection were recently proposed in the literature for application in BRs [247,248,249,250,251,252,253,254,255,256,257]. The innovative combination of droplet microfluidics and optical detection methods was proposed by Adams and coworkers for rapid measurements of glucose levels in the µBR systems [247]. For sensor application, a human hepatocarcinoma cell line was cultivated in a µBR for 10 days, and the system for optical detection, based on fluorescence, was used for measurements in droplet samples from the culture. The proposed solution has shown a fast response time in the range of glucose level 0–12 mM with a limit of detection of 0.2 mM.

Novel developments in the field of ECBs for cell culture applications propose enzyme immobilization and a combination of sensing technology with nanomaterials and microfluidic manipulation of small amounts of samples. **Enzymatic ECBs** for cell nutrients and metabolites can be generally divided into three categories. The first category utilizes enzymes to catalyze reactions, which generate by-products such as H_2_O_2_, that are further either oxidized or reduced at an appropriately polarized electrode [184]. The second type relies on the same concept, though the redox reaction happens with an additional redox pair as a mediator (e.g., osmium mediator) [258]. Firstly, a biological substance is oxidized on the sensor surface producing H_2_O_2_, which is further reduced by a second redox enzyme, usually a peroxidase, whereas the mediator (osmium(II)) gets oxidized [258,259]. The third category is characterized by a direct electron production through a redox reaction on the enzyme-deposited electrodes (mediator-free reaction) [260,261]. Moreover, a novel generation of **enzyme-free biosensors** is emerging where the analyte undergoes a redox reaction on a metal or metal oxide surface modified with various nanoparticles, nanosheets and nanoarrays, giving a product that is measured [260]. This is advantageous as it eliminates the influence of environmental effects, such as pH and temperature, which affect the activity of enzymes.

For the detection of cell culture nutrients and metabolites, oxidase enzymes (such as glucose oxidase, lactate oxidase, glutamate oxidase, pyruvate oxidase) are incorporated into the sensing platforms. Regardless of the oxidase enzyme type, the basic principle for such sensors is the production of H_2_O_2_ in equimolar concentration to the analyte, which is eventually oxidized at a noble metal electrode (usually platinum). If the analyte concentration is high (e.g., glucose or lactate), the release of H_2_O_2_ would also be substantial, which may cause adverse effects on the cells. Therefore, if the spatial separation of the sensor from the cells is not feasible, it is necessary to include an additional membrane that contains catalase which mediates the decomposition of H_2_O_2_ to oxygen and water. This approach is common in the case of the microfluidic systems [184].

The methodology for surface modification of electrodes with enzymes typically involves the reaction with glutaraldehyde to crosslink the enzymes with proteins (such as bovine serum albumin) and/or polymers containing free amine groups. In addition, novel methodologies are being demonstrated, such as immobilizing enzymes (glucose oxidase) on SU-8 surfaces [249]. The attachment between SU-8 and the enzyme is enabled by binding unreacted epoxy groups of SU-8 to the free NH_2_ group of glucose oxidase. The so-called Smart SU-8 pillars were integrated into the µBR and used for the continuous measurements of glucose concentration. The sensitivity of the glucose sensor was 33 ± 11 nA/mM with the linear response in the range of 10 mM but the sensitivity decreased over the measured period of 49 days.

In addition, different optical sensors were recently proposed for continuous measurements in the BRs, and some of them are commercially available. The novel single-use sensor for online measurements of glucose and online glucose sensor in shake flasks were recently put on the market by PreSens [250,251]. Furthermore, an optical fiber sensor for glucose was developed by modifying the surface of a commercially available oxygen sensor (OXY-4 mini^®^ PreSens, Regensburg, Germany) with crosslinked glucose oxidase. The results of the sensor integrated into the BR showed good durability of the sensor for a period of 52 days, linear characteristics up to 20 mM and the sensor’s response time lower than 10 min [248].

A cost-effective solution by using screen-printed electrodes for glucose monitoring in the BRs was proposed by Tang et al. [252]. The novelty of this study is in introducing a nanomaterial polymer matrix of oxidized cellulose nanocrystals (CNCs) for the covalent immobilization of glucose oxidase through carbodiimide chemistry. The application of the sensor was performed by monitoring glucose consumption in the fibroblast cell culturing for 7 days. The proposed sensor, in comparison with sensors from the literature that are based on glutaraldehyde-mediated crosslinking of enzymes [253,254,255,256] showed higher sensitivity, better stability, and longer shelf-life. However, the main drawback of the proposed solution is in a narrow range of glucose level detection, between 0.1 mM and 2 mM.

Amperometric glucose biosensor construction using gold nanoparticles-mesoporous silica composite (GNPs-MPS) was reported by Bai et al. 2-aminoethanethiol was used as a cross-linker for the immobilization of IO_4_^−^—oxidized-glucose oxidase on a GNPs-MPS-modified Au working electrode [262]. The catalytic behavior of the biosensor was examined by cyclic voltammetry and amperometry. The as-prepared biosensor exhibited a fast response time (less than 7 s), a broad linear range of 0.02–14 mM, as well as high sensitivity, good long-term stability and reproducibility. Another work incorporating mesoporous silica into the construction of a biosensor is reported by Li et al. [263]. In their study, amino-functionalized mesoporous silica nanoparticles were synthesized to immobilize both platinum nanoparticles (PtNPs) and glucose oxidase (GOx), forming MSN-PtNP-GOx. The as-synthesized composite was simply dropped onto the glassy carbon electrode surface for working electrode functionalization and biosensor preparation. The composite showed high stability and reactivity for catalyzing H_2_O_2_ electro-reduction due to the high surface area of the composite and the large amount of PtNPs immobilized. The biosensor exhibited interference-free glucose determination in a wide linear range from 1 μM to 26 mM.

Ges et al. fabricated a microfluidic device for trapping and culturing single cardiac myocytes (SMCs) in sub-nanoliter volumes with an integrated glucose-sensing electrode to track the glucose consumption by SMCs [264]. This miniaturized planar glucose electrode system was produced by spin coating Pt electrodes on glass substrates with a glutaraldehyde/enzyme solution and a Nafion membrane. Glucose electrodes showed high stability over a time period of 8 weeks and a response time between 5 and 15 s.

A promising sensor for **glutamate detection** and quantification was developed by Batra et al. with the limit of detection (LoD) of 0.1 nM. Their enzymatic sensor consisted of an immobilized glutamate oxidase on an Au electrode modified with polypyrrole (PPy) nanoparticles on polyaniline (PANI) (composite film) [265]. PANI is a conductive and biocompatible polymer, whereas PPy nanoparticles (NPs) exhibit a high surface area and high porosity, which, when combined, ensures enhanced conductivity and quick electron-transfer rate. Further work by Batra et al. resulted in the development of another enzymatic sensor based on glutamate oxidase (GluOx) combined with ZnO nanorods (NR) and PPy electrodeposited on pencil graphite [266]. The PPy polymer was used for its semiconducting properties, whereas the one-dimensional ZnO NRs ensure fast electron transfer kinetics and provide a large surface area. The LoD of this sensor was very low at 0.18 nM.

Özel et al. reported on a glutamate oxidase biosensor based on mixed ceria and titania nanoparticles for the detection of glutamate in oxygen-depleted conditions [267]. Oxygen-rich ceria and titania NPs were dispersed within a semi-permeable chitosan-based membrane and co-immobilized with the enzyme glutamate oxidase on the surface of a Pt microelectrode. L-glutamic acid was measured amperometrically exhibiting one of the fastest response rates at 2 s [267]. Scoggin et al. fabricated an enzymatic glutamate microbiosensor in the form of a Pt-microelectrode array on a ceramic-substrate to detect cellular glutamate uptake [268].

Hernández-Ibáñez reported an ECB for the **detection of lactate** within embryonic cell culture media [269]. Miniaturization of the lactate biosensor was achieved using screen-printed disposable electrodes as electrochemical sensing platforms. Composite composed of chitosan and multi-walled carbon nanotubes served for the immobilization of the lactate oxidase enzyme. The sensor was found to exhibit a linear response towards lactate in phosphate buffer with LoD of 22.6 µM. Shimomura et al. fabricated a device for amperometric L-lactate detection based on a screen-printed carbon electrode containing cobalt phthalocyanine coated with lactate oxidase–mesoporous silica conjugate layer [270]. Lactate oxidase was immobilized into mesoporous silica (FSM8.0) using a polymer matrix of denatured polyvinyl alcohol. The response of the as-fabricated biosensor was linear in the substrate range of 18.3 to 1.5 mM with a response time of 90 s.

The application of multi-wall carbon nanotubes (MWCNTs) in the sensor structure was shown to enhance the sensitivity of enzymatic biosensors for lactate and glucose [257]. A nanostructured electrochemical sensor with electrodes modified with MWCNTs and adsorbed glucose or lactate oxidase was applied for monitoring lactate production and glucose uptake in the SN56 neuronal cell line for 48 h. The proposed amperometric enzyme-based sensor achieved a sensitivity of 27.7 µA mM^−1^cm^−2^ and a detection limit of 73 µM.

**Ammonia (NH_3_)** is a gas present in the atmosphere in low concentrations (sub-ppb levels) and is part of the nitrogen cycle. The detection of ammonia has become increasingly important due to its industrial relevance, particularly in the food industry, as well as the hazard it poses to human health and the environment [271]. Ammonia causes a reduction of growth rates and maximum cell densities in batch cultures, changes in metabolic rates, perturbation of protein processing [272,273] and impacts the glycosylation profile [274]. Hence, controlling ammonia levels is a common goal in optimizing large scale cell culture.

The ammonia in the liquid media is mostly present in the form of the ammonium ions, i.e., NH_4_^+^ ions (NH_4_^+^ + OH^−^ ↔ NH_3_ + H_2_O). Ammonium ions (NH_4_^+^) can typically accumulate to concentrations of 2 mM during batch cultures and such a concentration has been shown to inhibit cell growth significantly [275]. Ammonia gas (NH_3_) is usually measured using commercially available multi-functional analyzers [276] applied on daily off-line samples. There are certain commercially available real-time analyzers, mostly based on Raman spectroscopy due to their applicability in aqueous systems without sample preparation [277], however all of these are still intended for pharmaceutical applications. There are also commercial devices for the continuous water quality monitoring of ammonia. These in-line ammonia monitors are generally very expensive, complex, and labor-intensive instruments and as such are difficult to justify on a cost basis.

Furthermore, traditional analytical techniques for ammonia detection, such as gas chromatography [278,279,280], ion mobility spectroscopy [279], or mass spectrometry [280,281] are limited by their impracticality, slow response time, and large instrument size.

Based on the above, it is clear that a need for developing a sensitive, selective, and affordable (low-cost) ammonia sensing technology has risen, which would be convenient for routine sampling and detection of ammonia gas, as well as the eventual continuous monitoring of ammonia emissions [282,283,284,285].

A plethora of sensing methods for NH_3_ detection have been developed, but not all have been implemented in a commercially available device. The NH_3_-sensing methods are classified into three major categories—solid-state sensing methods (metal oxide-based sensors, and conducting polymer sensors), optical methods (optical sensors utilizing tunable diode laser spectroscopy), and other methods (electrochemical sensors, surface acoustic wave sensors, and field-effect transistor sensors) [283,285]. In addition, different sensors for dissolved ammonia detection are available on the market [284].

The ammonia sensor with improved sensitivity and a lower detection limit was proposed for detection in cell culture media by combining microfluidics and colorimetric NH_3_ detection [286]. The cost-efficient solution for an optofluidic sensor was made by the integration of optical components in microfluidic platforms made of PDMS and SU-8. The detection principle is based on total internal reflection and the optofluidic chip contains a waveguide channel with the sample and integrated optical fibers from opposite sides of the waveguide, connected with light source and spectrometer, respectively. The amount of NH_3_ was calculated according to absorbance measurements and commercial ammonia quantification kits. The proof-of-concept was carried out by sampling and monitoring the concentration of NH_3_ during culturing adherent benign prostatic epithelial (BPH-1) cells over 48 h. The results showed better sensitivity and a lower limit of detection than traditional methods with microplate readers. However, the described procedure for the detection of ammonia is not automatic, i.e., requires sampling, separate incubation with reagents and subsequent introduction into the detection chip.

Microfluidics also opens possibilities for designing different serially connected microfluidic chips, for performing different steps automatically in the process of selective ammonia detection from complex aqueous solutions. Zhou et al. [287] demonstrated this approach by converting ammonium ions to gaseous ammonia though a reaction with a strong base within the reaction chip, followed by selective ammonia diffusion within a gas diffusion chip to a separate microfluidic channel, which subsequently leads to optical ammonia detection through a reversible reaction with Zn-tetraphenylporphyrin in a separate detection chip. The authors used PDMS to construct a gas diffusion membrane, though other materials such as polypropylene [288], polytetrafluoroethylene [289] or polyvinylidene fluoride [290] can be applied for ammonia diffusion as well.

**Figure 3:**
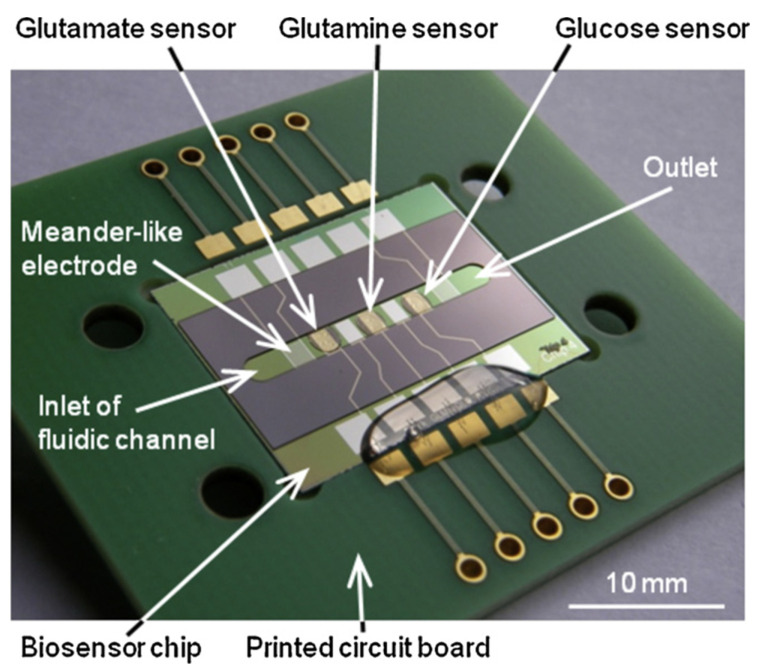
Photo of the biosensing chip comprising microfluidic channels and an array of amperometric enzyme sensors. The biosensor can simultaneously detect glucose, glutamate and glutamine, while the meander-like electrodes can detect temperature. Reprinted from [291]. Copyright (2020), with permission from Elsevier.

Importantly, microfluidics further enables the **detection of multiple analytes simultaneously (multiplex sensing)**. Figure 3 shows a microfluidic chip integrating amperometric enzyme sensors for the simultaneous detection of glucose, glutamate and glutamine in the cell culture [291]. The chip was constructed by attaching a Pt thin film on a SiO_2_ substrate through a Ti adhesion layer and crosslinking glucose oxidase, glutamate oxidase or glutaminase with glutaraldehyde in separate microfluidic compartments on the Pt surface. The biosensor chip was coupled to a flow-injection analysis (FIA) system for electrochemical characterization with the lower LoD at 0.05 mM for the glucose and glutamate sensor and 0.1 mM for the glutamine sensor. In order to prevent crosstalk among multiple sensors on a single chip, catalase membranes were incorporated to ensure H_2_O_2_ breakdown prior to entering the neighboring sensor [291].

Although most of the proposed solutions show good sensitivity and limit of detection, most sensors, even commercial ones—shown in Table 7, are not adapted for integration within BRs and application in cell culture for continuous monitoring.

On the other hand, most of the solutions proposed in the literature for applications in BRs remain without commercial implementation so far, mostly due to not meeting the requirements for BR application, such as stability under sterilization condition and long shelf life.

### 4.4. Photonic Sensors as Prospective Tool for Optical Monitoring of Cell Proliferation and Maturation

**Photonic sensors (PSs**) have been demonstrated as a promising tool for the analysis of liquids [292]. In the context of CM cultivation, this implies optical signal measurements based on refractive property changes occurring during cell culture, as well as spectral analysis [293]. Unlike conventional optical sensors, PS may be used to monitor the cell culture medium for weeks without the need to disturb the cell growth process, making use of easy access by the PS through the advantages of optical transducing and multivariate analysis of the output signal. This is particularly important for CM cultivation, which demands continuous online monitoring.

The integrated photonic sensors are an emergent technology rising alongside the technology of optical communication that uses similar concepts and approaches. The technology has initially been CMOS-based and implemented for the production of novel communication devices [294].

The main principle of detection in PSs is based on monitoring the changes in the evanescence field propagating in the vicinity of the optical waveguide surface. The refractive index is very sensitive to the changes of the environment on the media interface (e.g., glass/liquid) giving information both on the quantity of analyte and kinetic processes. There are a number of types of integrated planar optical waveguides based on different mechanisms of light signal processing. The most developed technologies are based either on Mach–Zehnder interferometers (MZIs), where the main response is generated by signal intensity changes or Young interferometers that use the changes in the interference pattern of reference and sensing channels of light propagation [295].

The main biorecognition approach of photonic sensors is based on assays similar to the immunoassays, where specific bioreceptors (antibodies, aptamers, DNA probes) are immobilized on the sensing side of the waveguide. The affinity-based conjugation of analyte and bioreceptor causes a refractive index change in the interface where the evanescent field is propagating, leading to a respective change in the light parameters that can be detected by a photodetector at the end of the waveguide.

Label-free photonic sensors technology based on planar optical waveguide and novel methods of signal processing have been developed during the last decades, leading to unprecedently low limits of detection [296].

Small molecules are always a critical problem in biosensing, requiring the use of sophisticated methods and technologies. The planar photonic biosensors can provide the analysis of small biomolecules using standard optical surfaces such as glasses without the need for complicated nanostructuring or modification. The current stage of development of the transducing mechanism in refractive-index photonic sensors has achieved the detection down to 10^−8^ RIU, providing the possibility of analyte traces’ detection with a concentration of pg/mL [295]. The planar optical CMOS-compatible technology is expected to move this limit further to fg/mL. A reduction in the amount of needed sample volume as well as decreasing the chemistry used for the assay will be additionally beneficial for the application in cellular agriculture.

Integrated optics allows for the manufacturing of dense arrays of sensors on the same chip enabling multiplexed analysis. Different types of materials, such as SiO_2_, Si, Si_3_N_4_, as well as various designs of sensing elements can be implemented. The CMOS-compatible processes such as ion implantation, chemical vapor deposition and lithographic techniques are commonly used for PS production. Recently, the optical elements based on polymers with the use of spin-coating and nanoimprint techniques were developed both to provide additional flexibility and decrease the production costs [297].

Nevertheless, silicon photonics is still a widely implemented technology for PS application because of the existing manufacturing infrastructures [298].

**Figure 4:**
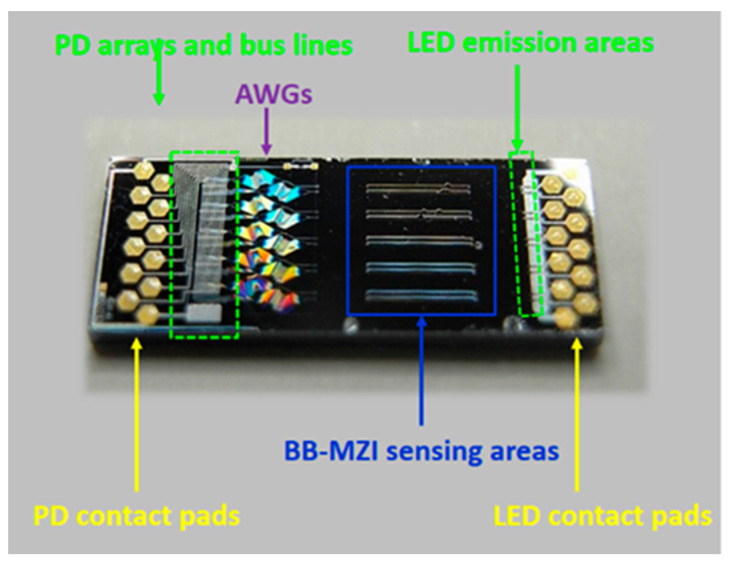
Photograph of the fully integrated spectroscopic chip: broadband Mach–Zehnder interferometers (BB-MZIs, blue rectangle), the arrayed waveguide gratings (AWGs, purple arrow). The light sources (LED) and photodiodes (PDs) (green rectangles) are connected to the electrodes. Adapted with permission from [299]. Copyright (2020) American Chemical Society.

The first truly portable PS-based biosensor was recently demonstrated by Misiakos and co-workers who integrated on the single chip the sensing element, spectrometers, lights sources and detectors [299]—Figure 4. The real-time detection of specific antibiotics in biological liquids was demonstrated. The fully spectroscopic silicon chip design provides a simple process of sensors’ cleaning and regeneration that insures the long-term stability and robustness of the device.

**Figure 5:**
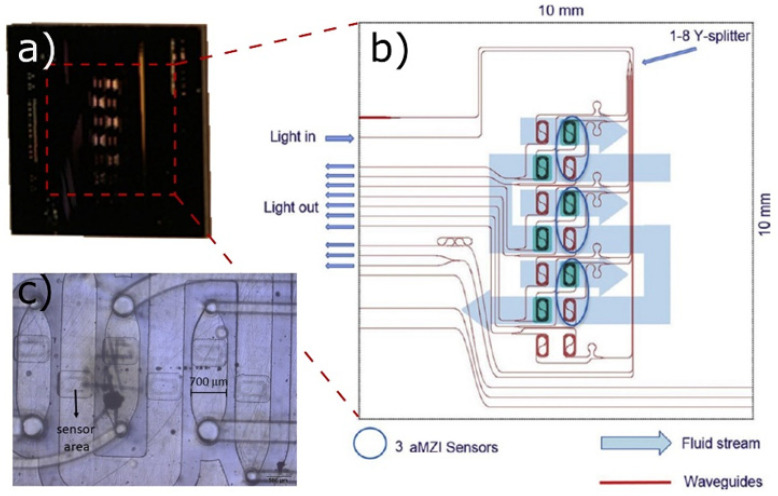
Multiplexed asymmetric Mach–Zehnder interferometer (aMZI) biosensor. (**a**) Photo of a chip; (**b**) layout scheme of the sensor chip: three sensors with an individual flow path and three with common flow path. (**c**) Close up image of three sensing elements, isolated by the soft acrylate sealing layer pressed above the aMZI. Adapted from [300]. Copyright (2020) with permission from Elsevier.

A multiplex signal analysis was recently shown by Fernández-Gavela et al. who used the asymmetric Mach–Zehnder interferometer (aMZI), integrated in lab-on-a-chip platform [300]—Figure 5. It includes up to six sensors situated in the microfluidic channels covering the waveguides. This ensures that each sensor can be used for multiple analytes analysis in a sample. The generic serum and antibiotics were used for the demonstration of reproducibility and stability of detection in the real environment. However, the long-term stability, particularly in the harsh environment, still needs to be further investigated.

Novel bioresorbable waveguides are discussed for clinical application using natural or synthetic polymers such as silk fibroin and polylactide-based materials (PLA, PLGA) [301]. The development of hybrid structures based on silicon and other inorganic and organic bioresorbable materials can be discussed as an additional option for the use in cultured meat process.

Another novel approach is based on “smart tattoo” sensors [302] enabling the in vivo monitoring of different chemical and physical processes in biological systems. The biosensor includes the fluorescent label in protective microspheres providing the possibility of biocompatible implantation into the tissue. The generated signal can be detected noninvasively by a custom optical fiber. Since this assay is isolated in a sphere, it is safe for use either when placed in the sensing module of a proliferation reactor or directly in the tissue construct in the perfusion bioreactor. The detection scheme based on remote optical fiber can be integrated into the bioreactor wall. Moreover, it is worth considering the concept of the “smart tattoo” as an additional level of quality control and even, potentially, as an anti-adulteration mark for the CM products.

In PS development, the novel materials and associated novel physical principles find their application for increasing sensor efficiency. For example, **graphene**, which is known for its unique properties stemming from the linear gapless energy band diagram and high charge carriers mobility, is discussed as a promising material for high-speed broadband photodetectors [303]. The interference of the light in waveguides integrated with graphene can increase both the efficiency of graphene photodiodes (PDs) and sensitivity of PS that was demonstrated using a different device concept such as a silicon-on-insulator [304] or photonic crystals [305]. Such types of graphene-coupled biosensors provide accurate and highly sensitive analyses of different reactions performed on a single chip [306,307]. However, the weak light-matter interactions of atomic-layer materials are challenging for the real-life application of graphene in optoelectronic devices [308].

In spite of significant developments concerning photonic biosensors, this is a field still in its infancy, particularly concerning the development of new algorithms of analysis, novel materials and new methods of functionalization with bioreceptors.

To summarize, the PS technology holds great potential for application for nutrients’ and metabolites’ detection in the CM research and prospective scale-up, since it is non-invasive, cost-efficient and able to detect small biomolecules in the medium, with extreme analytic sensitivity. All these attributes render PS highly advantageous for continuous online monitoring. However, there are still no commercial PS devices intended for application in CM production.

### 4.5. Longevity of the Sensing Elements in Real-Life Conditions

Concerning practical, real-life application, one needs to take into account the fact that during all phases of meat cultivation in different types of bioreactors, the conditions may be challenging for achieving efficient continuous and long-term sensing, as different unwanted materials from the complex reaction mixtures might cover the sensor surface, leading to the deteriorated performance of the sensors over time. Therefore, regular maintenance of the sensors, cleaning of their surfaces and sterilization between two cultivation processes are of great importance.

A suitable solution for the extension of the sensor’s reliability of measurements for some types of sensors, such as optical ones, can be the exploitation of the bioactive glass which has antimicrobial and antibiofilm activity [309,310]. Glass doped with copper, zinc, or silver showed good bioactive properties without ion release in the aqueous medium while doping with TiO_2_ and similar oxides proved to be an option for photocatalysis processes activated with visible light. However, as most sensors designed for implementation in bioreactors require a direct connection with a medium, a novel design of self-sufficient and autonomous sensors is urgently needed. These novel sensors should contain the capabilities of coupling to bioreactors, automatic sampling, separation of the desired analytes, and automatic cleaning or sterilization of the sensor surface before each run. This requires an additional fluidics system activated by a unique membrane pump, which connects the sensors to the bioreactor. The system can be designed as a closed-loop set-up, which returns the media to the bioreactor, or as an open one, where small amounts of the medium go to waste. Probably the most challenging step would be to realize an efficient separation of the specific analyte from the complex mixture. To achieve this, novel types of polymer-based membranes and/or in-built affinity chromatography may be viable solutions.

### 4.6. Image Detection and Recognition Techniques

The traditional meat industry has been implementing various imaging techniques for the quality and safety assessment of different types of meat [311]. However, when considering CM applications, the imaging technology concerns live-cell imaging techniques that ideally should allow the observation of internal structures and cellular processes in real-time and potentially can and should be combined with the above-described sensing options for enhanced CM bioprocess monitoring.

Using the imaging system enables the gathering of a wide array of data about the cell culture as viability, viable cell density, total cell density, cell health and cellular phenotype (morphology-related). Various cell monitoring imaging systems exist, including different types of microscopy systems (brightfield, phase–contrast, fluorescence). However, most of them require manual sampling or specifically designed chambers [312,313,314].

Live-cell imaging can be challenging since the process needs to be optimized for the particular assay readout, spectral compatibility, and signal-to-noise level. For reliable imaging results with live cells, it is recommended that the cells are maintained as closely as possible to the physiological temperature, pH, DO, and other conditions which they have during the cultivation process. This is where integration with the sensing systems is desirable.

One of the imaging systems designed to operate inside the incubator is MuviCyte [315]. It enables cell monitoring in various cell culture vessels (Petri dishes, flasks, microplates). However, it presents only a small platform and is not convenient for use in bioreactors.

Microfluidic platforms are a good option if one needs to combine live imaging with retrieving individual cells of interest [93,316].

For the bioreactor scale, a convenient option is to perform imaging using a shunt loop, as shown in Figure 2, where the medium and the cells enter the separate imaging module connected by the shunt loop. The sampled cells and medium can be either returned to the bioreactor chamber (proper sterility of the shunt needs to be maintained) or discarded. Efficient on-line shunt cell monitoring in real-time is carried out by the company Ovizio that implements 3D holography and analyzes detected signals by artificial intelligence, providing a number of different cell quality attributes and viability and viable cell density estimations [317]. Importantly, the shunt option is applicable for the microcarrier-based culture as well, as demonstrated by using Cytodex 1 MCs. The same method can be applied to any round, non-porous, transparent microcarriers [318].

## 5. Conclusions

This review has presented a wide spectrum of literature on various sensing technologies that may be applicable for the cultured meat (CM) bioprocess monitoring, complemented by the overview of the main tissue engineering aspects, such as scaffolding and bioreactors. Such a multidisciplinary approach is necessary in order to consider different views—those of the engineers, physicists and chemists fabricating the sensors and figuring out the options for integration of the sensors to the bioreactors and those of the tissue engineers and biologists who are concerned with maintaining cell viability and optimal metabolic parameters. Ultimately, all have the same goal—a sustainable, safe, cost-efficient cultured meat bioprocess, yielding a CM product with high quality and safety. The authors hope the examples and considerations presented and discussed here will help the readers of any background in the efforts to bring the cultivated meat to the market, while developing in the process various more advanced sensing options that may be utilized in other fields as well. 

## Figures and Tables

**Table 1:** Main types of commercially available bioreactors.

Type of Bioreactor	Volume	Phase of CM Cultivation	Type of Agitation/Medium Flow	Integrated Sensors	Ref.
Spinner flask	60–500 mL	Proliferation	Impeller-driven	n/a	[78,79]
Stirred tank	1–5 L–2 × 10^4^ L *	Proliferation	Impeller-driven	pH, dissolved oxygen and temperature control	[80]
				Air, N_2_, CO_2_ and O_2_, pressure, optical density, viable cells, exhaust gas composition, redox, weight for reactor	[81]
Rocking/wave	1–100 L *	Proliferation	Rocking motion-driven	pH and dissolved oxygen control	[82]
				internal floating filter (retains the cells in the bioreactor-filters only the media)	[83]
Perfusion bioreactors	up to 6000 L *	Differentiation/maturation	Perfusing medium through the scaffold	pH, temperature, automatic medium exchange, glucose measurement, mechanical and electrical cell stimulation	[84]
				bidirectional and interstitial perfusion, flow rate control	[85]

* The volume of the reactors used for commercial biomanufacturing bioprocesses other than cultured meat (CM).

**Table 2:** Commercially available temperature sensors.

Principle	Sensor	Temperature Range	Accuracy/Class	Ref.
Resistance sensors	Platinum	−200 to 1000 °C	offered in class F0.3 (0.12%), class F0.15 (0.06%) and F0.1 (0.04%)	[188]
	Nickel	−60 to 300 °C	6180 ppm/K (Nickel ND), 5000 ppm/K (Nickel NL), 6370 ppm/K (Nickel NJ), 6720 ppm/K (Nickel NA)	
	TSic	+10 to +90 °C	±0.5 K to ±0.1 K	
	United Electric Controls	−196 to 482 °C	RTP1 (std.) ± 0.12% RTP1A ± 0.06% RTP1AA ± 0.01%	[189]
Thermocouple	IST, Rosemount™	−40 to 750 °C	1.5 °C or 0.004 |t| *t* is in degrees Celsius.	[188,190]
	Krohne	−40 to 600 °C	±0.1% or ±0.15%	[189]
	Pyroscience, Burns	0 to 50 °C	±0.10 °C	[191,192]

**Table 3:** Commercially available pH sensors.

Principle	Sensor		Range	Accuracy	Ref.
Optical	Pyroscience	pH Sensor Spots	Different ranges available (4–6; 5–7; 6–8; 7–9; total scale)	±0.05 after 2-point calibration	[191]
		pH Flow Through Cell	Different ranges available (4–6; 5–7; 6–8; 7–9; total scale)	±0.05 after 2-point calibration	
		pH Sensor Cap for Under water devices	Different ranges available (4–6; 5–7; 6–8; 7–9; total scale)	±0.05 after 2-point calibration	
	PreSens Sensors	pH-1 SMA LG1	4.5–7	resolution: ±0.1 °C accuracy: ±1.0 °C	[198]
		Self-adhesive pH Sensor Spots SP-LG1-SA	4.5–7	resolution at pH = 7 ± 0.01 accuracy ±0.05/±0.10	
		Single-Use pH Flow-Through Cell FTC-SU-HP5-S	5.5–8.5	resolution: ±0.02 accuracy: ±0.05	
		Profiling pH Microsensor PM-HP5	5.5–8.5	resolution: ±0.01 accuracy at pH = 7 ± 0.1	
Electrochemical	pH Probes		Total scale	n/a	[197]
	Hygienic pH Probe for Sterile Applications		Total scale	n/a	[200]
	Bioreactor pH Probe		Total scale	Accuracy: ±0.1	[199]

**Table 4:** Commercially available oxygen sensors.

Principle	Sensor		Range/Accuracy	Ref.
Paramagnetic Cells Technology	Paramagnetic O_2_ Analyser		Different ranges available: 0–2%, 0–10%, 0–30%, 0–100%, 98–100% and 20–22%.	[203]
Optical	Mettler Toledo	Optical Dissolved Oxygen Sensors	8 ppb to 25 ppm with accuracy ±1%	[204]
	PreSens Oxygen Sensors	OXY-4 SMA (G3)	0–100% O_2_detection limit 15 ppb dissolved oxygen	[205]
		Self-adhesive Oxygen Sensor Spot SP-PSt3-SA	0–100% O_2_ Dissolved O_2_: 0–45 mg/L Accuracy ±0.4% O_2_ at 20.9% O_2_	
		O_2_ Flow-Through Cell FTC-PSt3	Dissolved O_2_: 0–45 mg/L ± 0.4% O_2_ at 20.9% O_2_	
Electrochemical	Polarographic Dissolved Oxygen Sensors		0–10.000 ppb Accuracy ± 1%	[204]

**Table 5:** Commercially available CO_2_ sensors.

Principle	Sensor		Range/Accuracy	Ref.
Optical	PreSens CO_2_ Sensors	CO_2_-1 SMA	range: 1–25% accuracy: ±0.06% at 2% CO_2_, ±0.15% at 6% CO_2_	[205]
		CO_2_ Sensor Spot SP-CD1	range: 1–25% accuracy: ±0.06% at 2% CO_2_, ±0.15 % at 6% CO_2_	
		CO_2_ Microsensor IMP-CDM1	range: 0.04%–5% CO_2_ accuracy: ±0.01% at 0.1% CO_2_, ±0.1% at 1% CO_2_	
Potentiometric	CO_2_ Sensor InPro5000i/12/120		range: 0.145–14.5 psig pCO_2_ accuracy: ±10	[211]

**Table 6:** Commercially available capacitive sensors for biomass. BR: bioreactor.

Sensor	Frequency Range	Capacity	Conductivity Range	Resolution	Type of BR	Ref.
Standard Remote Futura	50 kHz–20 MHz	0–400 pF/cm	1–40 mS/cm	Bacteria 2 × 10^9^ cells/mL for *Escherichia coli* Yeast or Animal cells 10^5^ cells/ml	Small bioreactors (up to 100 mL working volume)	[232]
Standard Futura	50 kHz–20 MHz	0–400 pF/cm	1–40 mS/cm	Bacteria 2 × 10^9^ cells/mL for *E. coli* Yeast or Animal cells 10^5^ cells/mL	Suitable for most BRs	[233]
BioPAT^®^ ViaMass	50 kHz–20 MHz	0–400 pF/cm	1–40 mS/cm	Yeast Bacteria Plant Cell	Suitable for single-use fermentation bags such as the Flexsafe^®^ RM	[231]
i-Biomass	n/a	0–700 pF/cm	0.5–100 mS/cm	10^5^ cell/mL for animal cells	Single-use BR	[234]

**Table 7:** Sensors for nutrients and metabolites used during cell culturing.

Glucose Sensors				
Principle	Structure	Glucose Concentration	Limit of Detection	Ref.
Optical	commercially available oxygen sensor that is coated with cross-linked glucose oxidase	0–20 mM	0.45 mM	[248]
Amperometric	SU-8 pillars with immobilized enzymes	0–12 mM	n/a	[249]
Amperometric	screen-printed sensor modified with cellulose nanocrystals	0.1–2 mM	0.004 mM	[252]
Electrochemical	nanocrystalline cellulose	1.0 to 20 mM	50 ± 10 µM	[253]
Electrochemical	zinc oxide nanoparticles on graphene–carbon nanotube	10 μM to 6.5 mM	4.5 (±0.08) μM	[254]
Electrochemical	three dimensional ordered macroporous self-doped polyaniline/Prussian blue bicomponent film	2 to 1600 μM	0.4 μM	[255]
Electrochemical	carbon nanotubes	0.073 to 4 mM	73 μM	[257]
Electrochemical	gold nanoparticles-mesoporous silica composite	0.02–14 mM	n/a	[262]
Electrochemical	glucose oxidase and platinum on mesoporous silica nanoparticles	0.001–26 mM	0.2 µM	[263]
Amperometric	enzyme electrodes	0–20 mM	n/a	[264]
Amperometric	enzyme-based sensors	0–20 mM	0.05 mM	[291]
**Glutamate Sensors**				
**Principle**	**Structure**	**Glutamate concentration**	**Limit of Detection**	**Ref.**
Amperometric	glutamate oxidase adsorpted on electrodeposited chitosan	20–352 μM	2.5 ± 1.1 μM	[258]
Amperometric	crosslinking of glutaraldehyde on platinum microelectrode	20–217 μM	6.5 ± 1.7 μM	
Amperometric	covalent immobilization of glutamate oxidase on polypyrrole nanoparticles/polyaniline modified gold electrode	0.02 to 400 μM	0.1 nM	[265]
Electrochemical	l-glutamate oxidase immobilized onto ZnO nanorods/polypyrrole modified pencil graphite electrode	0.02–500 μM	0.18 nM	[266]
Electrochemical	neurochemical probe	10–570 µM	6.3 ± 0.95 µM	[268]
Amperometric	enzyme-based sensors	0–10 mM	0.05 mM	[291]
**Lactate Sensors**				
**Principle**	**Structure**	**Glutamate Concentration**	**Limit of Detection**	**Ref.**
Electrochemical	chitosan/carbon nanotubes modified screen-printed graphite electrodes	30.4–243.9 µM	22.6 µM	[269]
Amperometric	carbon nanotube	0.028–2 mM	28 µM	[257]
Amperometric	screen-printed carbon electrode	18.3 μM–1.5 mM	n/a	[270]
**Ammonia sensors**				
**Principle**	**Structure**	**Ammonia Concentration**	**Limit of Detection**	**Ref.**
Optical	SU-8 microfluidic device	3–70 μM	2.5 μM	[286]
Conductivity	Lab-on-Chip with channel system	0–234 ppb	1.1 ppb	[288]
Electroosmotic	microfabricated electroosmotic pump coupled to a gas-diffusion microchip	0.25–5 mg/L	0.10 mg/L	[289]
Optical	microfluidic chip coupled with spectrophotometric method	Ammonium 0.2–50 mg/L	n/a	[287]
Optical	flow injection system coupled with spectrophotometric method	Ammonium 50–1000 μg/L	42 μg/L	[290]

## Data Availability

No new data were created or analyzed in this study. Data sharing is not applicable to this article.

## Abbreviations

ABS	acrylonitrile butadiene styrene
AC	alternating current
ADA	alginate di-aldehyde
AMR	antimicrobial resistance
aMZI	asymmetric Mach-Zehnder interferometer
Au	gold
AWG	arrayed waveguide gratings
bASCs	bovine adipose-derived stem cells
BPH-1	benign prostatic epithelial cells
BR	bioreactor
CA	cellular agriculture
CFD	computational fluid dynamics
CH_4_	methane
CM	cultured meat
CMOS	complementary metal oxide semiconductor
CNCs	cellulose nanocrystals
COVID-19	coronavirus disease-19
CO_2_	carbon dioxide
DNA	deoxyribonucleic acid
DO	dissolved oxygen
ECBs	electrochemical biosensors
ECM	extracellular matrix
EI	environmental impact
EU	European Union
FAO	Food and Agriculture Organization
FBS	fetal bovine serum
FDA	Food and Drug Administration
FIA	flow-injection analysis
FMIA	Federal Meat Inspection Act
FSANZ	Food Standards Australia New Zealand
GHG	greenhouse gas
GluOx	glutamate oxidase
GMO	genetically modified organism
GNPs-MPS	gold nanoparticles-mesoporous silica composite
GOx	glucose oxidase
HeLa	Henrietta Lacks
hMSCs	human mesenchymal stem cells
H_2_O_2_	hydrogen peroxide
H5N1	Hemagglutinin Type 5 and Neuraminidase Type 1
H7N9	Hemagglutinin Type 7 and Neuraminidase Type 9
IO_4_^−^	periodate
iPSCs	induced pluripotent stem cells
IR	infra-red
KCl	potassium chloride
LCA	life cycle assessment
LED	light-emitting diode
LoD	limit of detection
LTCC	low-temperature co-fired ceramic
MCs	microcarriers
MSCs	mesenchymal stem cells
MSN-PtNP-GOx	mesoporous silica nanoparticles-platinum nanoparticles-glucose oxidase
MWCNT	multi-wall carbon nanotubes
µBRs	microbioreactors
N_2_O	nitrous oxide
NDIR	non-dispersive infrared
NH_2_ group	amino group
NH_3_	ammonia
NIR	near-infrared
NPs	nanoparticles
NR	nanorods
PANI	polyaniline
PBE	population balance equations
PD	photodiodes
PDMS	polydimethylsiloxane
PEG	polyethylene glycol
PGA	polyglycolic acid
PLA	poly(L-lactic acid)
PLGA	poly(lactic-co-glycolic acid)
PMMA	poly(methyl methacrylate)
PPy	polypyrrole
PS	photonic sensors
Pt	platinum
PtNPs	platinum nanoparticles
PVC	polyvinyl chloride
RF	radio frequency
RIU	refractive index unit
SARS-CoV-2	severe acute respiratory syndrome coronavirus 2
SCs	satellite cells
SFA	Singapore Food Agency
Si	silicon
SiO_2_	silicon dioxide
Si_3_N_4_	silicon nitride
SMCs	single cardiac myocytes
SS	stainless steel
SU-8	“Structured by Uv”-“8 epoxy groups”
SUBs	single use bioreactors
Ti	titanium
US	United States
USDA–FSIS	US Department of Agriculture Food Safety and Inspection Service
ZnO	zinc oxide
2D	two-dimensional
3D	three-dimensional
3T3 cells	“3-day transfer, inoculum 3 × 10^5^ cells”
